# Effects of *cre1* modification in the white-rot fungus *Pleurotus ostreatus PC9:* altering substrate preference during biological pretreatment

**DOI:** 10.1186/s13068-018-1209-6

**Published:** 2018-07-27

**Authors:** Shahar Yoav, Tomer M. Salame, Daria Feldman, Dana Levinson, Michael Ioelovich, Ely Morag, Oded Yarden, Edward A. Bayer, Yitzhak Hadar

**Affiliations:** 1 Department of Plant Pathology and Microbiology, Robert H. Smith Faculty of Agriculture, Food and Environment, The Hebrew University of Jerusalem, Rehovot, 76100 Israel; 2 Flow Cytometry Unit, Life Sciences Core Facilities, Weizmann Institute of Science, Rehovot, 76100 Israel; 3 CelDezyner Ltd., 2 Bergman Street, Rehovot, Israel; 4 Department of Biomolecular Sciences, The Weizmann Institute of Science, Rehovot, 76100 Israel

**Keywords:** Biological pretreatment, White-rot fungi, *cre1*, Secretome, Decomposition of lignocellulose, CAZymes

## Abstract

**Background:**

During the process of bioethanol production, cellulose is hydrolyzed into its monomeric soluble units. For efficient hydrolysis, a chemical and/or mechanical pretreatment step is required. Such pretreatment is designed to increase enzymatic digestibility of the cellulose chains inter alia by de-crystallization of the cellulose chains and by removing barriers, such as lignin from the plant cell wall. Biological pretreatment, in which lignin is decomposed or modified by white-rot fungi, has also been considered. One disadvantage in biological pretreatment, however, is the consumption of the cellulose by the fungus. Thus, fungal species that attack lignin with only minimal cellulose loss are advantageous. The secretomes of white-rot fungi contain carbohydrate-active enzymes (CAZymes) including lignin-modifying enzymes. Thus, modification of secretome composition can alter the ratio of lignin/cellulose degradation.

**Results:**

*Pleurotus ostreatus PC9* was genetically modified to either overexpress or eliminate (by gene replacement) the transcriptional regulator CRE1, known to act as a repressor in the process of carbon catabolite repression. The *cre1*-overexpressing transformant demonstrated lower secreted cellulolytic activity and slightly increased selectivity (based on the chemical composition of pretreated wheat straw), whereas the knockout transformant demonstrated increased cellulolytic activity and significantly reduced residual cellulose, thereby displaying lower selectivity. Pretreatment of wheat straw using the wild-type PC9 resulted in 2.8-fold higher yields of soluble sugar compared to untreated wheat straw. The overexpression transformant showed similar yields (2.6-fold), but the knockout transformant exhibited lower yields (1.2-fold) of soluble sugar. Based on proteomic secretome analysis, production of numerous CAZymes was affected by modification of the expression level of *cre1*.

**Conclusions:**

The gene *cre1* functions as a regulator for expression of fungal CAZymes active against plant cell wall lignocelluloses, hence altering the substrate preference of the fungi tested. While the *cre1* knockout resulted in a less efficient biological pretreatment, i.e., less saccharification of the treated biomass, the converse manipulation of *cre1* (overexpression) failed to improve efficiency. Despite the inverse nature of the two genetic alterations, the expected “mirror image” (i.e., opposite regulatory response) was not observed, indicating that the secretion level of CAZymes, was not exclusively dependent on CRE1 activity.

**Electronic supplementary material:**

The online version of this article (10.1186/s13068-018-1209-6) contains supplementary material, which is available to authorized users.

## Background

Cellulose is the major component of plant cell walls that can potentially be converted to biofuel and other useful chemicals. Besides cellulose, the plant cell wall contains other polymers, such as hemicellulose and lignin, which encompass the cellulose and together creating a rigid, recalcitrant structure, which is difficult to hydrolyze enzymatically [1, 2]. In addition to its function as a mechanical barrier that restricts the accessibility of cellulolytic enzymes, lignin can also bind proteins in a nonspecific manner, leading to a decrease in the effective concentration of the cellulolytic enzymes available for the hydrolytic process [3–6]. Cellulose itself is an intractable molecule, containing crystalline regions, reinforced by inter- and intramolecular hydrogen bonds. To efficiently hydrolyze lignocellulosic biomass and to efficiently utilize the energy contained in cellulose, a pretreatment step is invariably required. The overall outcome of this pretreatment is the increase in digestibility of the available sugars. An optimal pretreatment strategy would enrich the cellulosic fraction, increase accessibility of the cellulose to the hydrolytic enzymes, remove or modify the lignin, and would be environmental friendly (i.e., minimized use of energy and chemicals along with reduced environmental wastes) [7, 8].

Numerous pretreatments, including physical (such as milling, steam explosion, and microwave), chemical (alkaline, acidic, etc.) or various combinations have been discussed in the literature [9]. Biological pretreatment, in which lignin is degraded or modified by microorganisms, has also been suggested. Biological pretreatment is considered to be environmentally friendly, since this approach exhibits low-energy demand/low carbon footprint and environmentally harmful chemicals are not used [9]. In contrast to the physical/chemical pretreatments used today, biological pretreatment does not require special facilities, since extreme heating or high pressures are not involved. Thus, biological pretreatment can also decrease the overall bioethanol production costs. Finally, unlike most other pretreatments, biological degradation of lignin is not accompanied by accumulation of compounds inhibitory towards yeast (such as furans), which may interfere with the downstream fermentation step [7, 8, 10–12]. On the other hand, biological pretreatment has some disadvantages, such as the requirement of long incubation periods and consumption of cellulose by the microorganisms during pretreatment [7, 10, 12, 13]. An efficient biological pretreatment strategy should thus result in a high ratio of lignin-to-cellulose degradation, with minimal cellulose loss.

Breakdown and consumption of lignocellulosic biomass by the fungus is mediated by a set of CArbohydrate-Active enZymes (CAZymes. Carbohydrate-Active Enzyme database, http://www.cazy.org/) [14]. The carbohydrates are decomposed by enzymes such as glycoside hydrolases (GHs), copper-dependent lytic polysaccharide monooxygenases (LPMOs), carbohydrate esterases (CEs), and pectate lyases (PLs), while the lignin modification is mediated by another set of enzymes, such as peroxidases, laccases, aryl alkyl oxidases (AAO), oxidoreductases, and copper radical oxidases (CRO). It is expected that altering the ratio between the lignin-modifying and the carbohydrate-decomposing enzyme activities in the secretome could influence the cellulolytic activities and utilization of cellulose by the fungus, and, consequently, the content of the residual lignocellulose biomass. The addition of metal salts (MnSO_4_ and CuSO_4_), phenolic and aromatic compounds (ferulic acid, xylidine, veratric acid, vanillic acid, cinnamic acid, and guaiacol) was found to increase selectivity by either increasing ligninolytic activity or by inhibiting cellulase activity [15–19]. Extracted natural cellulose inhibitor (NCI) from leaves of *Vitis vinifera* was also found to improve cellulose recovery and delignification, during biological pretreatment of paddy straw with *P. florida* [20].

Selecting a suitable microorganism for pretreatment is also a matter of consideration. A microorganism that demonstrates high ligninolytic capabilities but only minimal levels of cellulose consumption will be most appropriate for biotechnological applications. An extensive range of wood-decaying white-rot *Basidiomycota* (including *P. ostreatus*) were sequenced, enabling genomic, transcriptomic, and proteomic analysis of their plant cell wall-decaying enzymes [21–24]. Accumulating data show that there is no apparent genetic explanatory factor or phenotype pattern which would explain the preference for either lignin or cellulose degradation. In fact, based on comparative genomics of white-rot fungi, it was suggested that the functional diversity of these fungi could be better described as a continuum [25, 26] and could be also temporal (depend on growth kinetics) or related to the substrate chemical composition [21, 27]. A wide range of white-rot fungi, including *Irpex lacteus*, *Pycnoporus cinnabarinus*, *Phanerochaete chrysosporium*, *Trametes versicolor*, *Ceriporiopsis subvermispora*, *P*. *eryngii,* and *P. ostreatus* (alone or in consortia) were extensively studied for their potential for biological delignification [12, 28–36]. While evaluating the effect of rice straw pretreatment using several white-rot fungi, *P. ostreatus* was the most promising, demonstrating relatively selective removal of Klason lignin [35]. *P. ostreatus* was also found to be one of the preferred white-rot fungi for pretreatment of wheat straw, compared with 19 other tested white-rot fungi [36]. In a previous study conducted in our lab, we have genetically modified *P. ostreatus* PC9 to overexpress the ligninolytic enzyme versatile-peroxidase (VP1), independent of its natural Mn^2+^-dependent repression. The mutant displayed significantly higher levels of lignin mineralization, in vitro dry matter digestibility, and neutral detergent fiber digestibility [37].

Carbon catabolite repression (CCR) is a biochemical mechanism that down-regulates the synthesis of enzymes required for degradation and utilization of a carbon source when a preferred and easily metabolizable one (such as glucose) is available [38]. MIG1/CreA/Cre1 is zinc-finger transcription-factor homologs, conserved in most fungal species [38–45]. They were found to act as repressors in the CCR pathway in *Saccharomyces cerevisiae* as well as in some lower multicellular fungi, such as *Aspergillus niger*, *A. nidulans*, *Trichoderma reesei,* and *Neurospora crassa*. In the two latter species, CRE-1 was found to down-regulate cellulolytic genes and Δ*cre1* mutants exhibited increased cellulolytic activity. Transcriptome and secretome analyses of the Δ*cre1* mutants suggested that the regulation mechanism of CRE1 is complex, affecting both up- and down-regulation of genes and goes beyond CCR [43].

With the aim of altering the ratio between lignin and cellulose decomposition, the *cre1* gene of *P. ostreatus* PC9 was either overexpressed or disrupted in the current study. The resultant *cre1* overexpression (OE*cre1*) and *cre1* deletion (KO*cre1*) demonstrated different levels of secreted-cellulase activities. By performing secrotome analyses, we identified 138 CAZymes secreted by the *P. ostreatus* PC9 and transformants, and determined the changes that occur in their expression following manipulation of *cre1*. In addition, the respective cell wall composition after pretreatment and the biological pretreatment efficiencies of the wild-type PC9 and both genetically modified transformants were investigated. Manipulation of *cre1* expression altered the secreted CAZyme composition and the resultant pretreatment. Nevertheless, the parental PC9 demonstrated the highest pretreatment efficiency in terms of soluble sugar yield after pretreatment and hydrolysis.

## Results

### Production of cre1 transformants and their cellulolytic activity

**Fig. 1:**
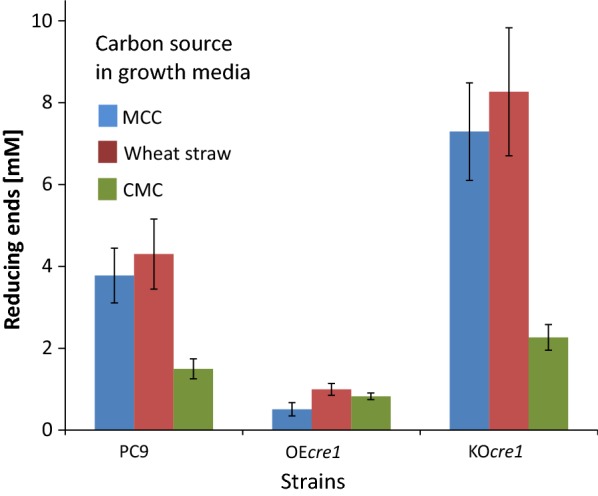
Secreted cellulolytic activity of PC9, OE*cre1,* and KO*cre1* transformants, grown on minimal media containing different carbon sources. The three stains were grown for 7 days on minimal media, containing MCC (blue), wheat straw (red) or CMC (green) as sole carbon sources. Similar volumes of supernatant were transferred to a PASC solution, and samples were incubated overnight. Cellulolytic activity in the spent media was monitored by measuring the concentrations of the released reducing sugars

With the aim of down-regulation of the cellulolytic activity of *P. ostreatus*, its *cre1* homolog was targeted for overexpression. The *P. ostreatus* PC9 *cre1* homolog was identified using BLASTP analysis against the *N. crassa* CRE1 protein (gene ID NCU08807). PleosPC9_1 126548 demonstrated the highest similarity (*E* value of 7 × 10^−30^, which is at least 13 orders of magnitude smaller than the other detected candidates) and the highest coverage (39% of the sequence). Previously, we have shown that the *β*-*tubulin* promoter is effective for driving overexpression in *P. ostreatus* [37]. Thus, it was chosen to induce constitutive overexpression of *cre1* by transformation of the wild-type strain (PC9) with the *β*-*tubulin* promoter-based TMS17 cassette (Additional file 1: Figure S1a). After the initial isolation step, the transformants were grown without selection. Four transformants showing similar linear growth rates to that of the wild-type on YMG-agar medium were further grown on minimal medium containing 2% MCC. Their secreted cellulolytic activity was measured by applying the supernatant fluids from the growth media on phosphoric acid swollen cellulose [46] and measuring the released soluble reducing sugars (Fig. 1 and Additional file 2: Figure S2). Transformant OE7 (designated herein OE*cre1*) presented the lowest activity (compared to PC9 and to the other transformants) and hence was selected for this study.

To up-regulate cellulolytic activity, the *cre1* homolog was targeted for knockout. The knockout was accomplished using the TMS18 gene replacement cassette, targeted for homologous recombination at the *cre1* gene locus (Additional file 1: Figure S1b), to transform the carboxin-resistant *P. ostreatus* PC9 Δ*ku80*, with the use of an additional selectable marker (Hygromycin). The two transformants revealed similar linear growth rates compared to that of the wild type, one of which (KO1, designated herein KO*cre1*) demonstrated higher activity relative to the PC9 and, hence, was selected for this study (Fig. 1 and Additional file 2: Figure S2).

**Fig. 2:**
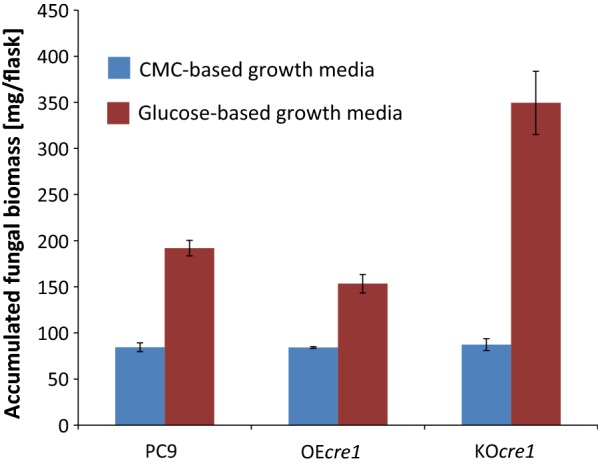
Fungal biomass accumulation. The three stains (wild-type and two mutants) were grown for 7 days on minimal media, containing CMC (blue) or glucose (red) as carbon sources. The accumulated fungal biomass was oven-dried and weighed

The two transformants and PC9 were further grown on liquid minimal medium with CMC (2%) or finely milled wheat straw (3%) as a carbon source. A similar pattern of lower cellulolytic activity for OE*cre1* and higher cellulolytic activity for KO*cre1* was observed in each of the growth media (Fig. 1). Fungal biomass accumulation was also measured, while grown on minimal medium with either CMC or glucose. PC9, OE*cre1,* and KO*cre1* demonstrated similar fungal biomass accumulation while grown on CMC-containing media. On the other hand, while grown on glucose-containing medium, KO*cre1* accumulated almost twofold the amount of biomass, when compared to the others (Fig. 2).

### Mass spectrometry analysis of secretomes revealed differentiation in CAZyme composition

**Fig. 3:**
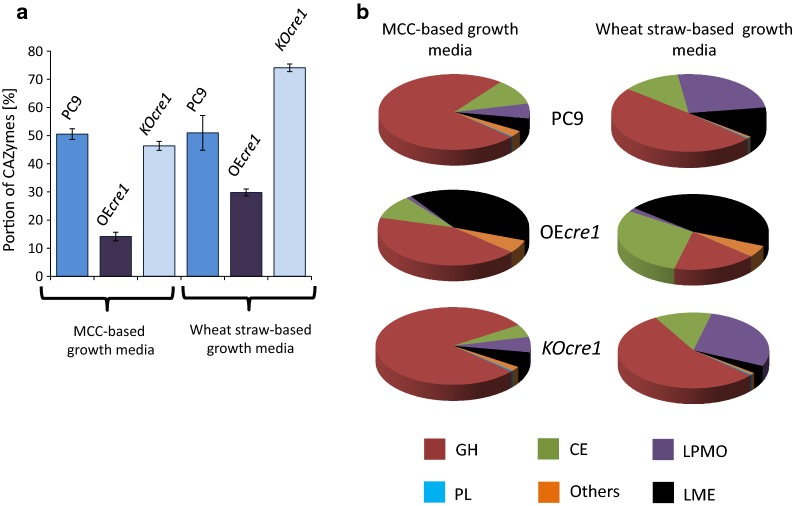
Relative portion of CAZymes. The three secretomes of the wild-type PC9 and the two transformants were analyzed by LC–MS/MS, and the results were compared to the JGI genome database of *P. ostreatus* PC9 v1.0, and further compared to the *P. ostreatus* PC9 v1.0 CAZyme database (for identification and classification of detected CAZymes). **a** Relative portion of CAZymes within each secretome. **b** Relative portion of each group (polysaccharide lyases [PL]; glycoside hydrolases [GH]; carbohydrate esterases [CE]; lytic polysaccharide monooxygenases [LPMO]; lignin-modifying enzyme [LME]; others) within the secreted CAZymes in the different secretomes

To further investigate the effects of altering *cre1* expression, the CAZyme composition in the fungal secretomes was analyzed by mass spectrometry. The fungi were grown on both MCC and finely milled wheat straw-containing media, and the secretomes were analyzed by LC–MS/MS. Peptide sequences from the MS data were compared to the JGI genome database of *P. ostreatus* PC9 v1.0 [21]. The CAZymes within the secretomes were identified by the JGI *P. ostreatus* PC9 v1.0 CAZyme database (https://genome.jgi.doe.gov/mycocosm/annotations/browser/cazy/summary;BrRlbi?p=PleosPC9_1). Only proteins with at least two identified peptides (of which at least one of them is unique) were considered for further analysis. Measured intensities were normalized using the intensity-based absolute quantification (iBAQ) method, enabling the comparison of different proteins in a given sample. Altering the expression of *cre1* influenced the expression levels of CAZymes. The relative portion of CAZymes within the secretome [sum of CAZymes iBAQ intensities·100/total secretome of iBAQ intensities] was lower in secretomes of OE*cre1,* grown on either MCC or wheat straw (14.1 and 29.7%, respectively). The secretome of KO*cre1* yielded a higher relative portion of CAZymes when grown on wheat straw (74.1%) but not on MCC (46.3%), compared to that of the wild-type PC9 secretome (50.5 and 50.9% for MCC and wheat straw, respectively (Fig. 3a).

To further analyze the composition of CAZymes from each secretome, the detected proteins were classified into six groups: lignin-modifying enzymes (LMEs), glycoside hydrolases (GHs), carbohydrate esterases (CEs), lytic polysaccharide monooxygenases (LPMOs), polysaccharide lyases (PLs), and others, according to their annotation in the CAZy database. The relative portion of each group in the different secretomes is presented in Fig. 3b. The OE*cre1* CAZY enzyme composition was comprised of a higher portion of LME and lower portion of carbohydrate-degrading enzymes (GHs, LPMOs, and PLs), while grown on either wheat straw or MCC. Such changes in the CAZyme composition can affect the ratio of lignin-to-carbohydrate decomposition. The KO*cre1* secretome had lower representation of LMEs and slightly higher GHs, LPMOs, and PLs (compared to the wild-type PC9), when grown on wheat straw, indicating potential reduced lignin degradation in comparison to carbohydrate decomposition. On the other hand, the abundance of LMEs and LPMOs in KO*cre1* grown on MCC was similar to that of the PC9, indicating the role of the carbon source in the regulatory effect of *cre1*.

**Fig. 4:**
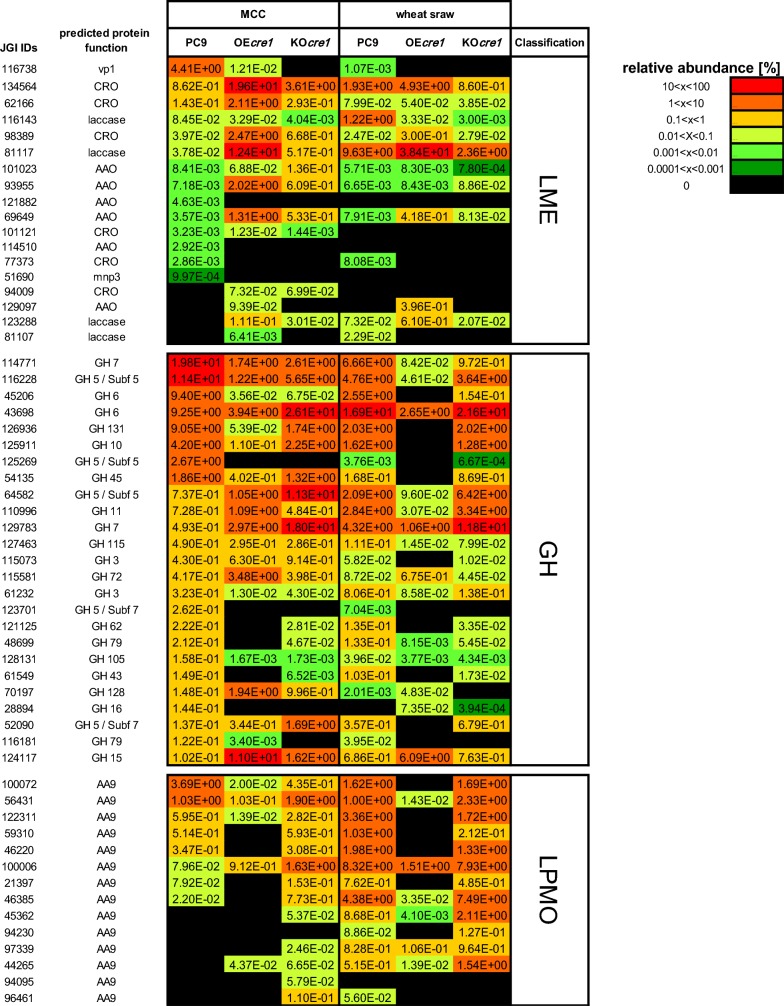
Secretome composition: relative abundance of individual enzymes within the secreted CAZymes of the wild-type (PC9), overexpression (OE), and knockout (KO) transformants, grown on either cellulose (MCC) or wheat straw, was calculated. The relative abundance of LMEs, LPMOs and top 25 abundant GHs is presented in the figure. The relative abundance of all CAZymes is presented in Additional file 3: Figure S3. Relative abundances were rated by a colored scale. The JGI ID number and annotated function for each enzyme are shown in the figure. AAO, aryl alkyl oxidase; AA9, auxiliary activity Family 9 (LPMO); CRO, copper radical oxidase; mnp3, manganese peroxidase 3; vp1, versatile-peroxidase

Next, we analyzed the relative abundance of individual CAZymes detected in the MS data. The effect of altering *cre1* expression on the relative abundance of the LMEs, LPMOs, and the 25 most abundant GHs within the six different secretomes is summarized in Fig. 4. The effect of altering *cre1* expression on all detected CAZymes is presented in Additional file 3: Figure S3. Different enzymes were affected differently by *cre1* overexpression (statistical analysis for each protein is available at Additional file 4: Table S1). For example, a decrease in the relative abundance of 29 GHs was observed in the secretome of OE*cre1* grown on MCC, when compared to that of PC9. However, *cre1* overexpression resulted in an increase in the relative abundance of 19 GHs. Among the 18 LMEs detected in the secretome of OE*cre1* grown on MCC, the relative abundance of 5 LMEs was decreased, while the relative abundance of 10 other LMEs was increased under the same growth conditions. *cre1* knockout also resulted in inconsistent regulation effects. The relative abundance of 19 GHs was decreased in the secretome of KO*cre1* grown on MCC, while the relative abundance of 16 GHs was increased under the same growth conditions. In this context, only enzymes that revealed an increase or decrease of at least one order of magnitude (or newly detected/undetected enzymes) were considered.

Although we initially assumed that OE*cre1* and KO*cre1* would demonstrate inverse patterns of expression, where enzymes whose abundance decreased in the OE*cre1* secretome would be present in higher quantities in the KO*cre1* secretome, our findings did not substantiate this behavior. For example, among 13 detected LPMOs in the wheat straw secretomes, the relative abundance of 11 was lower in the OE*cre1* secretome than in the wild type, but none was increased in the secretome of KO*cre1*. Moreover, some enzymes, such as 123701 (GH5), 114771 (GH7), 128131 (GH105), 116738 (VP1), and 77373 (Glyoxal oxidase), exhibited a decrease in relative abundance (or were not detected) in both of the transformants. Others, such as 124117 (GH15), 98024 (GH3), 100398 (GH7), 126650 (GH74), 93955 (AAO), and 69649 (AAO), revealed increases in their relative abundance in both transformants when grown on MCC (Fig. 4 and Additional file 3: Figure S3). The regulatory effect of *cre1* was also substrate-dependent. For example, the above-mentioned decrease in the relative abundance of the 11 LPMOs (with no increase of any LPMO) was observed only in the secretome of OE*cre1* grown on wheat straw. However, when OE*cre1* was grown on MCC, only 6 LPMOs showed a decrease in relative abundance, while that of 2 other LPMOs was increased.

### Biological pretreatment of wheat straw revealed different selectivity and, consequently, different levels of soluble sugars released after hydrolyses

**Fig. 5:**
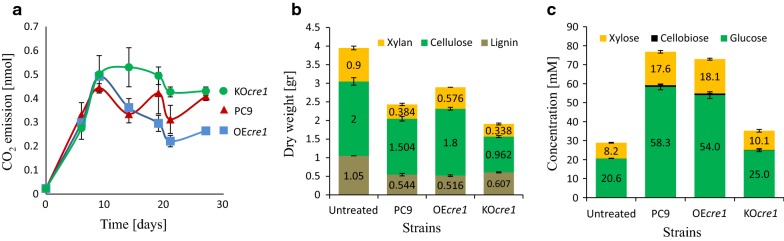
Biological pretreatment of heat straw by the wild-type PC9 and the two transformants. **a** Carbon dioxide emission during pre-pretreatment. **b** Chemical composition of pre/untreated wheat straw. **c** Soluble sugar concentration after the hydrolysis of the pre/untreated wheat straw using Cellic^®^ CTec2

To evaluate the efficacy of the biological pretreatment, five cultures of each fungus were grown on wheat straw for 35 days. Growth was monitored by measuring respiration (carbon dioxide emission) during the incubation period (Fig. 5a). Similar levels of respiration were observed in all the cultures during the first 9 days of the incubation period. Afterwards, however, an increase or decrease in CO_2_ emission was measured in the KO*cre1* and OE*cre1* cultures, respectively. Only 52% of the initial dry weight of wheat straw was recovered after pretreatment with KO*cre1*, compared to 64% in the case of PC9-treated wheat straw, and 72% when OE*cre1* was used. Chemical composition of the pretreated biomasses revealed different lignin-to-cellulose content ratio. Pretreating the straw with KO*cre1* resulted in a 42% reduction of lignin and 51.9% reduction of cellulose, compared to 48% lignin loss and 24.8% cellulose loss in the PC9-treated samples and 50.8% lignin loss and only 10% cellulose loss in the OE*cre1*-pretreated samples (Fig. 5b).

In a sequential step, the pretreated wheat straw samples were hydrolyzed with a commercial cellulolytic cocktail (Cellic^®^ CTec2), and the released soluble sugar concentrations were measured (Fig. 5c, Additional file 5: Figure S4). Biological pretreatment of wheat straw with PC9 increased the detectable glucose and xylose concentrations almost three- and twofold, respectively, compared to the untreated sample. Similar results (2.6-fold glucose concentration and 2.2-fold xylose concentration) were measured in the case of the OE*cre1*-pretreated wheat straw hydrolysate. The efficiency of KO*cre1* pretreatment, however, was much lower (Fig. 5c). Interestingly, only negligible amounts of cellobiose and no xylobiose were detected, indicating the presence of *β*-glycosidases in the commercial cellulase/hemicellulase cocktail. Although CTec2 is commonly used as a standard for degradation of lignocellulosic biomass, the lack of knowledge regarding its contents poses a general hindrance to this and other similar research efforts [47].

## Discussion

Cellulose from lignocellulosic biomass is a promising resource for alternative energy (e.g., bioethanol), as well as for other biochemicals. Thus, the process of converting lignocellulosic biomass into soluble fermentable sugars is of great interest for the biotechnological industry. Lignin acts as a physical barrier that restricts the accessibility of the carbohydrate chains of the plant cell wall to the catalytic enzymes. Lignin is also known for its non-productive adsorption (and thus inhibition) of the hydrolytic enzymes [1–6]. Hence, a substrate pretreatment step is usually necessary to remove or displace lignin, to increase the efficacy of lignocellulosic biomass hydrolysis. White-rot fungi possess a promising potential for biological pretreatment, due to their unique ability to completely and efficiently break down lignin [12, 13, 36, 48–51]. Indeed, several white-rot fungi have been analyzed for their potential as lignocellulose decomposing agents and have been demonstrated to be able to reduce lignin content and increase hydrolysis efficiency [12, 28–36].

One of the major drawbacks of biological pretreatment is the consumption of cellulose by fungi, at the expense of glucose yield [7, 10, 12, 13]. A fungus that can degrade lignin with only minimal cellulose consumption would mitigate this shortcoming and increase the overall efficiency of bioethanol production, and thus can be defined as a selective [52, 53]. To this end, the potential of *P. ostreatus* PC9 and two mutants, modified in their CAZyme expression pattern to pretreat wheat straw, was investigated. As the lignocellulosic biomass is degraded outside the cells, the composition of the secreted enzymes, namely, the ratio between secreted lignin-modifying and carbohydrate-degrading enzymes, can affect the level of residual cellulose. The expression of these enzymes is regulated by CCR. CRE1, a conserved regulator in the CCR pathway, has been previously found to regulate cellulolytic genes in several ascomycetes, such as *A. niger*, *N. crassa,* and *T. reesei* [38–44]. In addition, homologues to CRE1 were found in 27 of the 31 basidiomycete genomes assessed [54]. Yet, no functional studies regarding the effect of CRE1 on the regulation of cell wall degrading enzyme was conducted in the basidiomycetes until now [55].

In view of the above, we hypothesized that a CRE1 homologue may also have a conserved role as a transcriptional regulator of carbon metabolism in the basidiomycete *Pleurotus*. Thus, two transformants were modified to either overexpress or eliminate expression of the *cre1* gene (termed OE*cre1* and KO*cre1*, respectively). Many studies have been conducted in fungi within the context of overproduction of cellulases, where the expression of CRE1 was eliminated or reduced [41, 43, 44, 56]. The purpose of the current study, however, was to explore the role of *Pleurotus* not as a cellulase producer but as a biological pretreatment agent. Consequently, unlike most previous studies, we examined the function of *cre1* overexpression in addition to its deletion.

OE*cre1* and KO*cre1* exhibited decreased or increased levels of secreted cellulolytic activity, respectively, while grown on either soluble cellulosic substrate (CMC), insoluble cellulosic substrate (MCC) or on a lignocellulosic substrate (wheat straw), thereby demonstrating the role of *cre1* repression in *P. ostreatus* (Fig. 1). Interestingly, OE*cre1* was able to grow on cellulosic substrates despite overexpression of *cre1*, consistent with the previous observations, showing that basal expression levels of cellulolytic genes are not repressed by *cre1* [43]. Similar levels of accumulated fungal biomass were observed in all three fungi, when grown on CMC. However, increased fungal biomass accumulation was observed when the KO*cre1* was grown on a glucose-based medium. Likewise, the growth pattern reported for the Δ*cre1* mutant of *N. crassa* was similar to that of the wild type when grown on CMC but different when grown on glucose [39]. Glucose is known to regulate carbon catabolite enzymes via the *cre1* repression pathway [57–60]. Thus, we expected that *P. ostreatus* that lacks *cre1* would grow differently on glucose-based media. Since the differences in the secreted cellulolytic activity are not applicable in such glucose-containing media, our observation supports the contention that CRE1 regulates functions beyond CCR, including the rate of glucose assimilation, and can influence cell growth [43].

The degradation of lignocellulosic biomass by fungi is carried out via the collective activity of secreted enzymes. The secretome composition, in addition to cellulolytic activity levels, is, therefore, of great interest, since changes in secretome composition can influence the efficiency of biological pretreatment. Indeed, MS analysis of the secretome is a commonly used method for investigating the cell wall degrading machinery of fungi [61–65]. The availability of the genome sequence of the monokaryotic *P. ostreatus* [21] enables us to perform MS analysis of its secretome [48, 62, 66]. The wild-type and *cre1*-transformants were grown on MCC and wheat straw (as sole carbon sources) and their secretome compositions were analyzed. The three fungi revealed differences in the percentage of CAZymes within the secretome (Fig. 3a), as well as differences in the composition of CAZyme groups within each fungus (Fig. 3b). The secretome of OE*cre1* grown on wheat straw exhibited a higher proportion of LMEs and lower abundance of carbohydrate-degrading enzymes (GHs, LPMOs, and PLs). In contrast, the secretome of KO*cre1* contained less LMEs and a slightly higher proportion of carbohydrate-degrading enzymes. The changes in the compositions of the secretomes are consistent with the observed changes in the composition of the pretreated wheat straw, demonstrating increased/decreased ratios of cellulose-to-lignin content in the OE*cre1*/KO*cre1*-treated wheat straw (respectively, Fig. 5b). Our results thus highlight the effect of altering *cre1* expression not only on the levels of secreted cellulolytic activity (an important parameter regarding cellulase production) but also on the potential to act as a biological pretreatment agent.

The effect of modifying *cre1* expression on the individual CAZymes was further investigated (Fig. 4). An extended range of CAZYmes was affected by *cre1* alteration: The relative abundance of approximately 70 and 55% of the detected CAZymes in the secretome of OE*cre1* and KO*cre1* (respectively) grown on MCC was either decreased or increased by at least one order of magnitude. While grown on wheat straw, 68 and 33% of the detected CAZymes in the secretome of OE*cre1* and KO*cre1* (respectively) was either decreased or increased. Interestingly, OE*cre1* and KO*cre1* did not demonstrate opposite regulatory response of CAZyme expression. Some proteins were less abundant in both transformants, regardless of whether *cre1* was overexpressed or inactivated. Conversely, other proteins were highly expressed in both. Some were affected only in one of the transformant. These results can indicate an extensive and complicated regulatory pathway involving *cre1* and/or the possible presence of overlapping/complementary regulatory pathways. Indeed, such complimentary regulation pathways of cellulose production have been reported for some ascomycetes. For example, cellulase expression in *N. crassa* was reported to be repressed by both CRE1-dependent CCR and by the glucose-sensing/metabolism transcription factor COL26, and a synergistic effect between the two regulation pathways was suggested [59]. In addition to repression, cellulase gene expression was also reported to be upregulated, *inter alia* by transcription factors CLR1 and CLR2 [67, 68]. Positive regulation of cellulase expression was also reported for other ascomycetes such as *A. niger, T. reesei*, *A. aculeatus,* and *A. nidulans* [67, 69, 70]. Moreover, the crosstalk between the two regulatory systems is currently not well understood [59]. Such complimentary regulation pathways can partially compensate for mutagenesis of *cre1* and might provide a possible explanation for our observations.

CRE1 affected the various CAZymes differently. Namely, the relative abundance of some genes was increased, whereas the relative abundance of others was decreased in each of the transformants. These observations can indicate a role for CRE1 not just as a repressor, but as a regulator that can also increase expression. These results are in accord with the previous reports which identified genes that showed decreased expression levels in a Δ*cre1* mutant (namely positively regulated by CRE1) in both *T. reesei* and *N. crassa* [39, 43] (including genes that were annotated as members of carbohydrate metabolism pathways). In this context, Portnoy et al. [43] suggested that *cre1* should be considered a master regulator (with up- and down-regulatory effects) rather than a repressor. It should be noted that OE*cre1* was manipulated via non-homologous integration, which also might be a possible source of the differences between the wild-type PC9 and the OE*cre1* transformant (e.g., neighboring effects).

The secretome of PC9 was previously analyzed when the fungus was grown on glucose, poplar wood, and wheat straw-containing media [66]. Our results highlight several similar trends in the secretome of PC9 grown on wheat straw samples, such as (i) the significant role of copper radical oxidases (CROs), rather than the aryl-alcohol oxidases (AAO) among the hydrogen peroxide-providing enzymes and (ii) the significant role of laccases [specifically lac10 (81117)], rather than the oxidoreductases (VP1 and MnP3) as the most abundance lignin-modifying enzymes.

Microorganisms are known to respond to available carbon sources and to change their secreted CAZymes accordingly [38, 42, 66, 71–74], e.g., via the CCR pathway [38, 39, 42, 43, 75]. Indeed, the secretome of PC9 has been found to change in response to different carbon sources [62, 66]. For example, the relative abundance of laccases, oxydoreductases, and peroxidases was shown to increase when grown on wheat straw compared to growth in a glucose-based medium [66]. By comparing the secretome of PC9 grown on MCC to wheat straw (regardless of altering *cre1* expression), our results also demonstrate a similar influence of the carbon source. The relative portion of LMEs in the secretome of PC9 grown on wheat straw was higher than that of cells grown on MCC, demonstrating “differentiation” towards the wheat straw (where LMEs are required). Interestingly, the preference of laccases as the most abundant lignin-modifying enzymes was observed only in the wheat straw samples but not in the MCC samples where VP1 displayed higher relative abundance than laccases. Since CRE1 mediates regulation of CAZyme expression in response to the available carbon source, it logically followed that *cre1* alteration would also be manifested differently when grown on different carbon sources. Indeed, the effect of modifying *cre1* expression on several CAZymes was found to be carbon source-dependent. For example, the relative abundance of 115073 (GH3) was decreased only in the secretome of OE*cre1* grown on wheat straw (where it was undetected) but not when grown on MCC (where no decrease was found). A similar trend was found for 52090 (GH5/Subf7). In general, it should be noted that only secreted soluble proteins were analyzed in the current work. Thus, the fact that some proteins were undetected (Fig. 4 and Additional file 3: Figure S3) does not necessarily mean that they were not expressed. The inability to detect a protein might also be related to substrate binding.

To investigate whether the changes in the secretome composition affect selectivity of lignin-to-cellulose degradation and the biological pretreatment efficiency of the mutants, the fungi were used to treat wheat straw. PC9 demonstrated the highest glucose yield after enzymatic hydrolysis (280%, compared to the untreated wheat straw). In comparison to PC9, KO*cre1* exhibited increased CO_2_ emission during pretreatment, a lower ratio of cellulose-to-lignin content and only a modest increase in the glucose yield compared to that of untreated wheat straw (121%). OE*cre1* showed decreased CO_2_ emission, which slightly increased the ratio of cellulose-to-lignin content and a glucose yield of 260% (slightly lower than that observed for PC9). It may thus be concluded that knockout of *cre1* can reduce selectivity and pretreatment efficiency. Nevertheless, overexpression of *cre1* can increase selectivity, but cannot improve the pretreatment efficiency.

Several fungi (alone or in a consortium), including *P. ostreatus*, were previously investigated for their biological pretreatment capabilities, demonstrating a wide range of lignin removal and increased hydrolysis yield upon treatment of woody and non-woody lignocellulosic biomass [12, 33, 35]. In the current work, the parental *P. ostreatus* PC9 was the most efficient, demonstrating 48.1% lignin removal and an increased hydrolysis yield of 2.8-fold after 35 days of pretreatment. Optimization process (time of growth, moisture percentage, additives in the medium, and other parameters) may increase pretreatment efficiency [34, 35]. Indeed, in a preliminary calibration step, two pretreatment periods (namely, 19 and 35 days) were compared. The latter (35 days) resulted in higher soluble sugar concentration (1.5- to 2-fold) after hydrolysis (Additional file 5: Figure S4), thus demonstrating the influence of pretreatment length on efficiency. For more efficient pretreatment using *P. ostreatus*, an extensive calibration process should be conducted.

## Conclusions

Increased lignin degradation, accompanied by reduced cellulose decomposition, can mitigate a major disadvantage in the biological pretreatment process of lignocellulosic biomass—i.e., the consumption of cellulose by the fungus. In the current paper, the expression of the gene encoding the transcriptional regulator CRE1 of the white-rot fungus *P. ostreatus* PC9 was manipulated, changing the secreted cellulolytic activity levels, the secretome composition, the selectivity of lignin degradation, and the efficiency of biological pretreatment. Knockout of *cre1* led to increased secreted cellulolytic activity, decreased selectivity of lignin degradation, and lower glucose yields. Overexpressing *cre1* led to decreased secreted cellulolytic activity, slightly increased selectivity of lignin degradation, but failed to generate higher glucose yields compared to the wild-type. Thus, changing *cre1* expression was found to be a suitable approach for altering substrate preference or for genetic engineering of overproduction of *P. ostreatus* cellulase, but not suitable for engineering a superior biological pretreatment organism. The regulation effect of the *cre1* homolog on secreted CAZymes in the basidiomycete *P. ostreatus* provided only a partial answer to its role in fungal biology.

## Methods

### Fungal and bacterial growth conditions

*Pleurotus ostreatus* monokaryon PC9 (Spanish-Type Culture Collection accession number CECT20311), which is a protoclone derived by dedikaryotization of the commercial dikaryon N001 (Spanish-Type Culture Collection accession number CECT20600) [76], and its derivative homokaryon Δ*ku80* (carboxin-resistant, designated 20b) [77] were used throughout this study. *Escherichia coli* JM109 cells (Promega, Madison, WI, USA) were used for the standard cloning procedures according to the manufacturer’s protocol. The fungi were grown and maintained on GP agar medium (2% [w/v] glucose, 0.5% [w/v] peptone (Difco), 0.2% [w/v] yeast extract (Difco), 0.1% [w/v] K_2_HPO_4_ (J.T Baker), 0.05% [w/v] MgSO_4_·7H_2_O (J.T Baker), and 1.5% [w/v] agar [78]. For the various characterization assays, the fungi were also grown on minimal media (DDW supplemented with 0.4% [wt/vol.] yeast extract [Difco] and a sole carbon source (carboxymethylcellulose [CMC, Sigma-Aldrich] or microcrystalline cellulose PH 101 [MCC]/glucose/finely milled wheat straw). Agar plates and liquid media were inoculated by GP agar disks (5-mm diameter) of mycelium obtained from the edge of a young colony and positioned at the center of the petri dish or spread in the flask, respectively. Cultures were incubated at 28 °C in the dark. When mentioned, the fungicide carboxin (Sigma-Aldrich) and the antibiotic hygromycin B (Alexis Biochemicals) were added to final concentrations of 2 mg/l (50% lethal dose [LD_50_] = 0.16 mg/l) and 100 mg/l (LD_50_ = 7 mg/l), respectively.

### Mycelial linear growth

The mycelial linear growth rate was determined by measuring the position of the advancing mycelial front (leading hyphae) in a solid YMG culture (1% [wt/vol.] glucose, 1% [wt/vol.] malt extract [Difco], 0.4% [wt/vol.] yeast extract [Difco], 1.5% [wt/vol.] agar). Agar disks of mycelium obtained from the edge of a young colony (at least three replicates for each) were placed in the center of a new YMG-agar plate, and the disks were incubated at 28 °C in the dark. After 7 days, and before the leading hyphae arrived to the plate edges, the radius of the colony was measured.

### Fungal biomass accumulation

Liquid minimal media (50 ml), containing 1% glucose or 2% CMC, were inoculated with the different stains (four disks, 0.5 cm each). Cultures were grown with shaking (120 rpm) for 6 days. Culture biomass accumulation was measured as dry weight (oven-dried to a constant weight at 65 °C).

### Nucleic acid manipulations

Molecular manipulations were carried out according to the standard protocols as described by Sambrook and colleagues [79]. Genomic DNA was extracted with the DNeasy Plant Mini Kit (Qiagen, Valencia, CA, USA) from culture biomass first ground under liquid nitrogen with mortar and pestle. Nucleic acid concentration and purity measurements were performed using a NanoDrop-2000 apparatus (Thermo Scientific, Waltham, MA, USA). PCR was performed in an Eppendorf Mastercycler Gradient Thermocycler, using Phusion High-Fidelity PCR Master Mix (Finnzymes, Vantaa, Finland), with the primers listed in Additional file 6: Table S2. Isolation and purification of DNA fragments from agarose gel or PCR amplification were performed using the Wizard SV Gel and PCR Clean-Up System (Promega). Cloning into plasmids was performed using the pGEM-T Vector System II (Promega). Plasmid DNA was purified using the QIAprep Spin Miniprep Kit (Qiagen). DNA endonuclease restriction and ligation were performed using restriction enzymes and T4 DNA Ligase (Fermentas).

### Construction of the cre1 overexpression and cre1 replacement cassettes

The candidate *P. ostreatus* PC9 *cre1* homolog was identified in the JGI genome database (http://genome.jgi.doe.gov/PleosPC9_1/PleosPC9_1.home.html, [21]) using BLASTP analysis against *N. crassa* CRE1 protein (gene ID NCU08807; http://fungi.ensembl.org/Neurospora_crassa, [80]). The protein with the highest similarity (gene ID 126548) was defined as the CRE1 homolog, and its coding region (scaffold 6:85499–88547) was used in this study. To construct the *cre1* overexpression cassette, the promoter and terminator regions of *β*-*tubulin* [81] and the coding sequence (CDS) of *cre1* were amplified from genomic DNA, using primers SacII-btubPF and cre1F-btubPR, btubPR-cre1F, and cre1R-SphI, respectively (Additional file 1: Figure S1a, Additional file 6: Table S2). The resulting amplicons were fused using the double-joint (fusion) PCR technique [82] and then ligated into plasmid pTM1 [83] using the *Sph*I and *Sac*II restriction sites to produce a conjunct *cre1* overexpression and carboxin-resistance (*Cbx*^*r*^) cassette designated TMS17 (Additional file 1: Figure S1a), which was cloned to produce plasmid pTMS17. To construct the *cre1* replacement cassette, the flanking DNA (2 kb 5′ and 3′) of *cre1* CDS was amplified from genomic DNA and the hygromycin B-resistance gene (*Hyg*^*r*^) from plasmid pTMS14 [77] using primers cre1PF and hygF-cre1PR, cre1PR-hygF and cre1TF-hygR, and hygR-cre1TF and cre1TR for the 5′ flank, 3′ flank, and *Hyg*^*r*^, respectively (Additional file 1: Figure S1b, Additional file 6: Table S2). The resulting amplicons were fused together using the double-joint (fusion) PCR technique [82] to produce a conjunct *cre1* replacement and hygromycin-resistance cassette designated TMS18 (Additional file 1: Figure S1b), which was cloned into the pGEM-T vector to produce plasmid pTMS18.

### Fungal transformation

Transformation was performed based on the PEG-CaCl2 protocol, previously adapted for *P. ostreatus* [77]. Either carboxin or hygromycin B were used as selection markers, and resistance was conferred via introduction of the carboxin-resistance cassette (*Cbx*^r^) or the hygromycin B-resistance cassette (*Hyg*^*r*^), respectively. Either plasmid pTMS17 or pTMS18 were used as the transforming DNA (Additional file 1: Figure S1a and b). Competent protoplasts were produced by digestion of 2 g (pelleted wet weight) of vegetative mycelium of *P. ostreatus* from YMG liquid culture with lytic enzymes. The lytic enzyme solution consisted of 2% w/v lysing enzymes from *Trichoderma harzianum* (Sigma-Aldrich, product number L1412) and 0.5 units/ml chitinase from *Trichoderma viride* (Sigma-Aldrich, product number C8241) in 0.5 M sucrose as an osmotic stabilizer. The protoplasts were washed [by centrifugation at 1670 rpm (450*g*), 8 min, 4 °C] in sorbitol-Tris-calcium (STC) solution (18.2% w/v sorbitol, 50 mM Tris–HCl, pH 8.0, 50 mM CaCl_2_, and 0.5 M sucrose) and adjusted to a final concentration of 5 × 10^7^ protoplasts/ml. Then, 2 ml of protoplasts were mixed with 100 μl of transforming DNA (300 ng/μl), 150 μl of heparin solution (Sigma-Aldrich, product number H4784) (5 mg dissolved in 1 ml of STC solution), and 300 μl of single-strand λ phage carrier DNA (Fermentas, product number SD0011, Glen Burnie, MD, USA) (500 μg/ml, after denaturation at 95 °C for 5 min and immediate transfer to ice). After 40 min of incubation on ice, 10 ml of polyethylene glycol–Tris–calcium solution (40% w/v PEG4000, 50 mM Tris–HCl pH 8.0, 50 mMCaCl_2_, and 0.5 M sucrose) was added, and the mixture was incubated for 20 min at room temperature. The mixture was then plated on solid YMG regeneration medium containing 0.5 M sucrose and allowed to regenerate overnight. Subsequently, a medium overlay containing either carboxin or hygromycin B was applied, obtaining a final concentration of 2 or 150 mg/l, respectively. Transformants were isolated after 10 days of incubation at 28 °C. Transformant stability was verified by three successive transfers (inoculated from the edge of a 10-day-old colony) to solid medium without the selection drug and then returning the transformant to solid culture conditions, in which the selective drug was present. Genomic integration of TMS17 and *cre1* gene disruption were confirmed by PCR, using the primers TMS17DF and TMS17DR, or cre1DF and cre1DR (respectively, Additional file 1: Figure S1a and b, Additional file 6: Table S2) on extracted DNA. Overexpression of *cre1* was also confirmed by real-time PCR analysis (Additional file 7: Figure S5).

### Measurement of secreted cellulolytic activity

Sterile 250 ml baffled Erlenmeyer flasks, containing 60 ml of minimal media with either 2% (w/v) MCC, 2% (w/v) CMC, or 3% (w/v) finely milled wheat straw, were inoculated with four disks of GP agar containing fresh mycelium. Flasks were incubated for 7 days with continuous shaking and then centrifuged. Supernatant fluids (125 µl) were added to 50 mM acetate buffer pH 5.0, containing 7.5 g/l phosphoric acid swollen cellulose (PASC, [46]) in a final volume of 0.5 ml, and the suspension was incubated at 50 °C for overnight. Released soluble sugar (reducing end) concentrations were analyzed by the dinitrosalicylic acid (DNS) method, as previously described [84]. Final soluble sugar concentrations were determined against a glucose calibration curve.

### Secretome preparation and mass spectrometry analysis

Growth media containing MCC or wheat straw (as described in the previous section) were centrifuged (10 min, 3400*g*), filtered (0.22 micron), and concentrated through a 5 kDa molecular weight cut-off device (Vivaspin 20 PES membrane, Sartorius AG, Germany). Concentrated samples were supplemented with cOmplete™ Protease Inhibitor Cocktail (Sigma-Aldrich, Rehovot, Israel) according to the manufacturer’s instructions and kept at minus 80 °C until use.

Secretome samples were dissolved in 8 M urea/100 mM ammonium bicarbonate, reduced by dithiothreitol at a final concentration of 2.8 mM (60 °C for 30 min) and modified with 8.8 mM iodoacetamide in 100 mM ammonium bicarbonate (30 min, room temperature, in the dark). The reduced, modified samples were then digested by the modified trypsin (Promega, Madison, WI) at a 1:50 enzyme-to-substrate ratio in 2 M urea, 25 mM ammonium bicarbonate (overnight, 37 °C) followed by a second digestion step (4 h). The tryptic peptide mixture was desalted using C18 tips (Ultra-Micro, Harvard), dried (speedvac) and re-suspended in 0.1% formic acid. The peptides were resolved by reverse-phase chromatography on 0.075 × 180-mm fused silica capillaries (J&W), packed with Reprosil-reversed phase material (Dr. Maisch GmbH, Germany). The peptides were eluted with linear 60-min gradient of 5–28%, 15 min gradient of 28–95% and 15 min at 95% acetonitrile with 0.1% formic acid in water at flow rates of 0.15 μl/min. Mass spectrometry was performed by Q Exactive plus mass spectrometer (Thermo Scientific, Waltham, MA) in positive mode using repetitively full MS scan followed by collision-induced dissociation (CID) of the ten most dominant ions selected from the first MS scan. The MS data were analyzed using MaxQuant software 1.6.0.16 (Cox and Mann [85]) vs. the JGI genome database of *P. ostreatus* PC9 v1.0 (http://genome.jgi-psf.org/PleosPC9_1/PleosPC9_1.home.html) [21]. MS data were also analyzed vs *S. cerevisiae* and *Triticum aestivum* proteomes (from the Uniprot database) to identify and omit proteins from non-fungal sources (namely the yeast extract or the wheat straw from the media). Peptide- and protein-level false discovery rates (FDRs) were filtered to 1% using the target-decoy strategy [86, 87]. The protein table was filtered to eliminate identifications from the reverse database and from common contaminants. The data were quantified by label-free analysis using the MaxQuant software, based on extracted ion currents (XICs) of peptides enabling quantitation from each LC/MS run for each peptide identified in any of the experiments. The mass spectrometry proteomics data have been deposited to the ProteomeXchange Consortium via the PRIDE partner repository [88] with the data set identifier PXD010100.

### Biological pretreatment of wheat straw

For evaluating the pretreatment, 1 g of wheat straw chips (< 2 mm) was supplemented with 4 ml DDW containing 73 µM Mn^2+^ in a 100 ml glass Erlenmeyer flask, autoclaved, inoculated with 3 grains of spawn produced on sorghum seeds, and incubated at 28 °C for 27 days. CO_2_ emission was measured during growth as was previously described [89]. Briefly, flasks were sealed with a gas-tight cap for 2 h. A 1-ml air sample was taken with a syringe and injected into a gas chromatograph (GC; Hewlett Packard 5890 gas chromatograph Series II, Santa Clara, CA, USA) equipped with an HP-PLOT Q GC Column (Agilent Technologies, Santa Clara, CA, USA) and a thermal conductivity detector. CO_2_ concentrations were calculated against a CO_2_ calibration curve (generated by samples with known CO_2_ concentrations). Results are presented as nmol CO_2_ emitted from the culture. After 35 days, each Erlenmeyer flask was supplemented with 5 ml of a solution containing 0.2 M acetate buffer, pH 5.0, 14 ml DDW, and 0.04 ml of Cellic^®^ CTec2 (Novozymes, China), and incubated at 50 °C for 42 h with continuous shaking, centrifuged (5 min, 17,000*g*), and the concentration of mono- and di-saccharides (glucose, cellobiose, and xylose) in the supernatant fluids was measured using Agilent Infinity 1260 high-pressure liquid chromatography (HPLC) equipped with Aminex HPX-87H 300/7.8 mm (Bio-Rad) column and 0.05 M H_2_SO_4_ as the mobile phase (flow rate of 0.6 ml/min). Final soluble glucose and xylose concentrations were determined against calibration curves. It should be noted that galactose (minor amounts) might be co-eluted with the xylose due to the nature of the column.

### Composition analysis of wheat straw samples

Triplicates of pretreated or untreated wheat straw (5 g each, 35 days of biological pretreatment) were independently analyzed. Samples were dried at 105 °C according to the NREL Standard Method LAP-001 [90]. Content of acid-insoluble lignin was determined according to the modified NREL Standard Method LAP-003 [91]. Briefly, samples were pre-hydrolyzed at 72 wt% sulfuric acid at 30 °C for 2 h. The concentration of sulfuric acid was then diluted with DDW to 4 wt%, and the sample was hydrolyzed in sealed crimp top serum bottles at 121 °C for 1 h. The resultant acidic dispersion of lignin was centrifuged (10 min, 3400*g*) and the sediment of lignin was washed with DDW until the pH reached 7, dried at 105 °C, and the weight of the lignin was measured.

The content of cellulose and xylan was analyzed using a modification of the standard method of NREL LAP-002 [92]. After determination of the lignin component, the hydrolysate was neutralized with CaCO_3_. The calcium sulfate formed was precipitated by centrifugation (10 min, 3400*g*). The concentration of monosaccharides (glucose and xylose) in the supernatant was measured using HPLC (as described above in the previous section), and the content of cellulose and xylan in the biomass was calculated.

## Additional files

**Additional file 1: Figure S1.** Genetic manipulation of *cre1* overexpression (OE*cre1*) and knockout (KO*cre1*) transformants. a. Strategy for overexpression of *cre1* in *P. ostreatus* (PC9). The *β*-*tubulin* promoter was fused to the *cre1* coding sequence (CDS) and ligated to the carboxin-resistance-conferring cassette (*Cbx*^*r*^) to produce the TMS17 cassette. Small arrows indicate the location of primers (Additional file 6: Table S2) used for construction and detection of the construct. PCR verification of the integration was performed using primers TMS17DF and TMS17DR (Additional file 6: Table S2) b. Strategy for gene replacement of *cre1* in *P. ostreatus* (Δ*ku80*, 20b). The hygromycin B-resistance-conferring cassette (*Hyg*^*r*^) was fused to 2 kb of 5′ and 3′ genomic DNA flanking *cre1* CDS to produce the TMS18 cassette. Wild-type *cre1* genomic region is illustrated below. PCR verification of *cre1* replacement was performed using primers CRE1DF and CRE1DR (Additional file 6: Table S2).

**Additional file 2: Figure S2.** Secreted cellulolytic activity of *cre1*-overexpressing or *cre1*-knockout transformants. *Pleurotus ostreatus* PC9 was genetically manipulated to either overexpress or knock out the *cre1* gene. The fungi were grown on minimal medium, containing 2% MCC, for 7 days. Secreted cellulolytic activity was measured by applying the supernatant fluids on phosphoric acid swollen cellulose and measuring the released soluble reducing sugars by the DNS method. The *cre1*-overexpressing transformant OE7 exhibited the lowest secreted cellulolytic activity and was thus selected for further analysis (termed herein OE*cre1*; red bar marked with black arrow). The *cre1*-knockout transformant showed the highest secreted cellulolytic activity and was thus selected for further analysis (termed herein KO*cre1*; green bar marked with black arrow).

**Additional file 3: Figure S3.** Relative abundance of individual CAZymes. Relative abundance [% of total secreted CAZymes] of the individual detected CAZymes was calculated, revealing the composition of secreted CAZymes in the different secretomes. Relative abundance of the CAZymes was rated by a colored scale as shown in the figure. The JGI ID number and the annotated function are shown in the figure.

**Additional file 4: Table S1.** Statistical analysis of mass spectrometry data. *P* values for the comparison between PC9 and the two transformants, for each identified protein in the secretomes, were determined by one-way ANOVA, following a post hoc Tukey’s HSD test. The comparison of PC9 grown on MCC vs wheat straw was determined using a two-tailed *T* test. Adjusted *P* values and adjusted values (after FDR correction) are presented in the table. The relative abundances (triplicate) for each protein are also included in the table.

**Additional file 5: Figure S4.** Hydrolysis of un/pretreated wheat straw. The wild-type PC9 and the two transformants were used as a biological pretreatment of wheat straw for 19 and 35 days. The pretreated and untreated straw samples were hydrolyzed by the commercial cellulase cocktail Cellic^®^ CTec2 (Novozymes), and the concentration of released soluble sugars was measured by the DNS method [84].

**Additional file 6: Table S2.** Oligonucleotides used in this study. The oligonucleotides that are used for either construction of cassettes or verification of the genomic integration of both OE*cre1* or KO*cre1* are detailed in the table. The location of the primer within the cassettes or in the genome is given in Additional file 1: Figure S1a and b.

**Additional file 7: Figure S5.** *cre1* gene expression analysis. PC9 and OE*cre1* were grown for 7 days on minimal media containing either CMC or glucose as a carbon source. Culture biomass was ground under liquid nitrogen with mortar and pestle, and RNA was purified by RNeasy Plus Mini Kit (Qiagen) according to the manufacturer’s instructions. cDNA was synthesized using the qScript cDNA Synthesis Kit (Quanta BioSciences, Gaithersburg, MD, USA). Quantification of transcript abundance was determined on an ABI StepOnePlus Real-Time PCR Sequence Detection System and software (Applied Biosystems), using Power SYBR Green PCR Master Mix (Applied Biosystems), with an annealing temperature of 63 °C, according to the manufacturer’s default operating procedures. The endogenous internal control gene used was *β*-*tubulin*. Primers tubF543 (5′-GTGCGTAAGGAAGCTGAGGG-3′) and tubR777 (5′-TGTGGCATTGTACGGCTCAAC-3′) were used to amplify a 201-bp amplicon from *β*-*tubulin*. Primers cre1F_2214 (5′-GTGGATTGGGCGGGTCGA-3′) and cre1R_2623 (5′-CATCCGTTCCCATGAGCGAT-3′) were used to amplify a 201-bp amplicon from *cre1*. Target gene transcript abundance is expressed relative to the levels of *β*-*tubulin* according to their Ct values 2 ^[(Ct *β*−tubulin) − (Ct *cre1*)]^, in arbitrary units.

## Abbreviations

AAO: aryl alkyl oxidases
CAZymes: carbohydrate-active enzymes
CE: carbohydrate esterase
CMC: carboxymethyl cellulose
CRO: copper radical oxidase
DDW: double-distilled water
iBAQ: intensity-based absolute quantification
GH: glycoside hydrolase
KO*cre1*: *cre1* deletion (knockout) transformant
LME: lignin-modifying enzyme
LPMO: lytic polysaccharide monooxygenase
MCC: microcrystalline cellulose
MS: mass spectrometry
NCI: natural cellulose inhibitor
OE*cre1*: *cre1* overexpressing transformant
PL: polysaccharide lyase
VP1: versatile-peroxidase 1
