# Supplementary material for: Effects of cre1 modification in the white-rot fungus Pleurotus ostreatus PC9: altering substrate preference during biological pretreatment
Source: Biotechnol Biofuels. 2018 Jul 27;11:212. doi: 10.1186/s13068-018-1209-6 (PMC6062969; doi:10.1186/s13068-018-1209-6)
Supplement: Supplementary file 1 — Additional file 1: Figure S1. Genetic manipulation of cre1 overexpression (OEcre1) and knockout (KOcre1) transformants. a. Strategy for overexpression of cre1 in P. ostreatus (PC9). The β-tubulin promoter was fused to the cre1 coding sequence (CDS) and ligated to the carboxin-resistance-conferring cassette (Cbxr) to produce the TMS17 cassette. Small arrows indicate the location of primers (Additional file 6: Table S2) used for construction and detection of the construct. PCR verification of the integration was performed using primers TMS17DF and TMS17DR (Additional file 6: Table S2) b. Strategy for gene replacement of cre1 in P. ostreatus (Δku80, 20b). The hygromycin B-resistance-conferring cassette (Hygr) was fused to 2 kb of 5′ and 3′ genomic DNA flanking cre1 CDS to produce the TMS18 cassette. Wild-type cre1 genomic region is illustrated below. PCR verification of cre1 replacement was performed using primers CRE1DF and CRE1DR (Additional file 6: Table S2). [file 13068_2018_1209_MOESM1_ESM.ppt]

## Slide 1
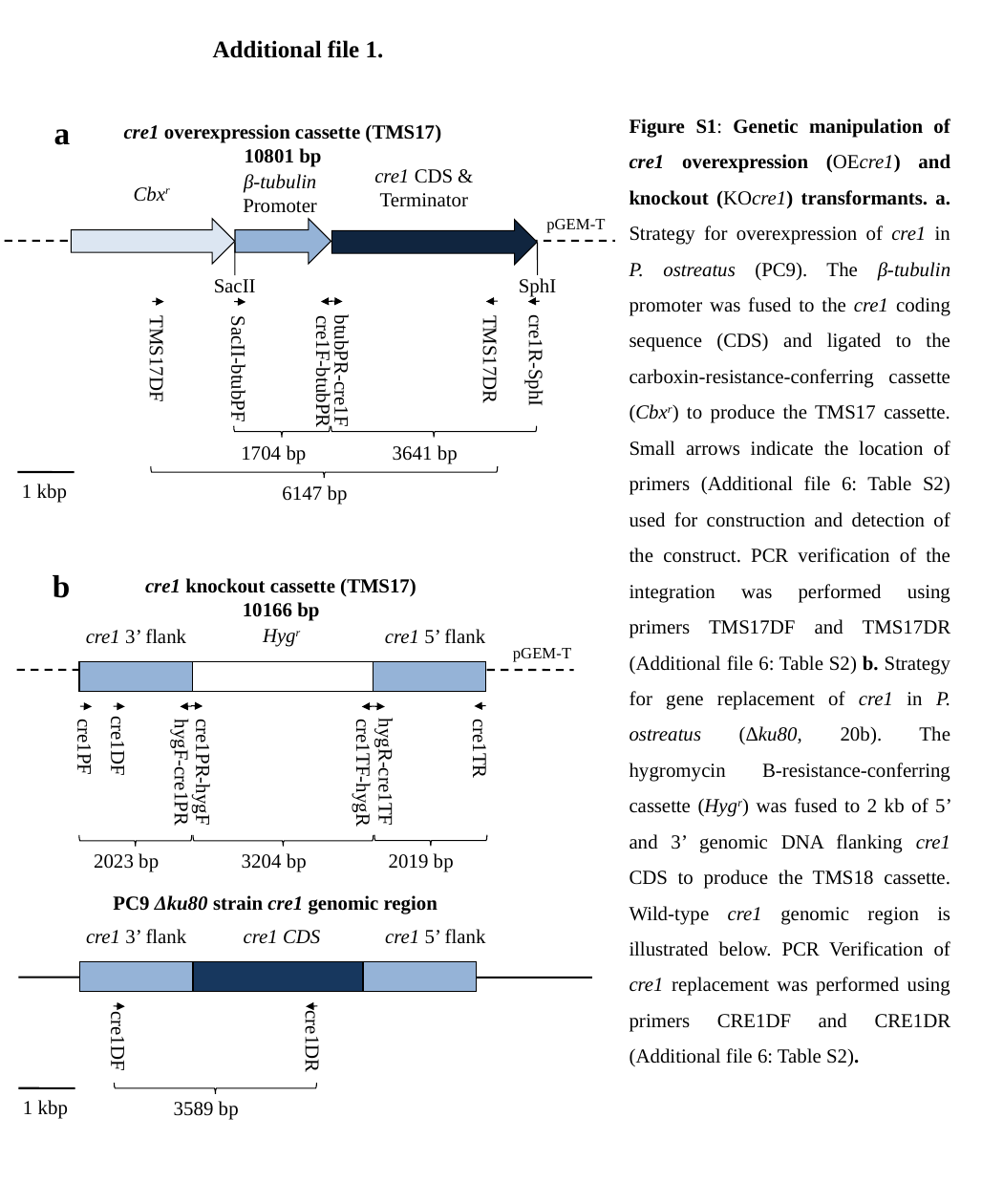

Additional file 1.
Figure S1: Genetic manipulation of cre1 overexpression (OEcre1) and knockout (KOcre1) transformants. a. Strategy for overexpression of cre1 in P. ostreatus (PC9). The β-tubulin promoter was fused to the cre1 coding sequence (CDS) and ligated to the carboxin-resistance-conferring cassette (Cbxr) to produce the TMS17 cassette. Small arrows indicate the location of primers (Additional file 6: Table S2) used for construction and detection of the construct. PCR verification of the integration was performed using primers TMS17DF and TMS17DR (Additional file 6: Table S2) b. Strategy for gene replacement of cre1 in P. ostreatus (Δku80, 20b). The hygromycin B-resistance-conferring cassette (Hygr) was fused to 2 kb of 5’ and 3’ genomic DNA flanking cre1 CDS to produce the TMS18 cassette. Wild-type cre1 genomic region is illustrated below. PCR Verification of cre1 replacement was performed using primers CRE1DF and CRE1DR (Additional file 6: Table S2).
a
cre1 overexpression cassette (TMS17)
10801 bp
cre1 CDS & Terminator
β-tubulin
Promoter
Cbxr
pGEM-T
SacII
SphI
cre1R-SphI
btubPR-cre1F
TMS17DR
TMS17DF
cre1F-btubPR
SacII-btubPF
3641 bp
1704 bp
1 kbp
6147 bp
b
cre1 knockout cassette (TMS17)
10166 bp
Hygr
cre1 3’ flank
cre1 5’ flank
pGEM-T
cre1DF
hygR-cre1TF
cre1TR
cre1PF
cre1TF-hygR
cre1PR-hygF
hygF-cre1PR
3204 bp
2019 bp
2023 bp
PC9 Δku80 strain cre1 genomic region
cre1 CDS
cre1 3’ flank
cre1 5’ flank
cre1DR
cre1DF
1 kbp
3589 bp
