# Supplementary material for: Effects of cre1 modification in the white-rot fungus Pleurotus ostreatus PC9: altering substrate preference during biological pretreatment
Source: Biotechnol Biofuels. 2018 Jul 27;11:212. doi: 10.1186/s13068-018-1209-6 (PMC6062969; doi:10.1186/s13068-018-1209-6)
Supplement: Supplementary file 2 — Additional file 2: Figure S2. Secreted cellulolytic activity of cre1-overexpressing or cre1-knockout transformants. Pleurotus ostreatus PC9 was genetically manipulated to either overexpress or knock out the cre1 gene. The fungi were grown on minimal medium, containing 2% MCC, for 7 days. Secreted cellulolytic activity was measured by applying the supernatant fluids on phosphoric acid swollen cellulose and measuring the released soluble reducing sugars by the DNS method. The cre1-overexpressing transformant OE7 exhibited the lowest secreted cellulolytic activity and was thus selected for further analysis (termed herein OEcre1; red bar marked with black arrow). The cre1-knockout transformant showed the highest secreted cellulolytic activity and was thus selected for further analysis (termed herein KOcre1; green bar marked with black arrow). [file 13068_2018_1209_MOESM2_ESM.docx]

**Additional file 2**

**Figure S2. Secreted cellulolytic activity of *cre1*-overexpressing or *cre1*-knockout transformants.** *Pleurotus ostreatus* PC9 was genetically manipulated to either overexpress or knock out the *cre1* gene. The fungi were grown on minimal medium, containing 2% MCC, for 7 days. Secreted cellulolytic activity was measured by applying the supernatant fluids on phosphoric acid swollen cellulose and measuring the released soluble reducing sugars by the DNS method [1]. The *cre1*-overexpressing transformant OE7 exhibited the lowest secreted cellulolytic activity and was thus selected for further analysis (termed herein OE*cre1*; red bar marked with black arrow). The *cre1*-knockout transformant showed the highest secreted cellulolytic activity and was thus selected for further analysis (termed herein KO*cre1*; green bar marked with black arrow).

1. Miller GL. Use of dinitrosalicylic acid reagent for determination of reducing sugar. Anal Biochem. 1959;31:426–8.
