# Supplementary material for: Effects of cre1 modification in the white-rot fungus Pleurotus ostreatus PC9: altering substrate preference during biological pretreatment
Source: Biotechnol Biofuels. 2018 Jul 27;11:212. doi: 10.1186/s13068-018-1209-6 (PMC6062969; doi:10.1186/s13068-018-1209-6)
Supplement: Supplementary file 3 — Additional file 3: Figure S3. Relative abundance of individual CAZymes. Relative abundance [% of total secreted CAZymes] of the individual detected CAZymes was calculated, revealing the composition of secreted CAZymes in the different secretomes. Relative abundance of the CAZymes was rated by a colored scale as shown in the figure. The JGI ID number and the annotated function are shown in the figure. [file 13068_2018_1209_MOESM3_ESM.docx]

**Additional file 3.**

**Figure S3. Relative abundance of individual CAZymes.** R**elative abundance [% of total secreted CAZymes] of the individual** detected CAZymes **was calculated, revealing the composition of secreted CAZymes in the different secretomes. Relative abundance of the CAZymes was rated by a colored scale as shown in the figure. The JGI ID number and the annotated function are shown in the figure.**
