# Supplementary material for: Effects of cre1 modification in the white-rot fungus Pleurotus ostreatus PC9: altering substrate preference during biological pretreatment
Source: Biotechnol Biofuels. 2018 Jul 27;11:212. doi: 10.1186/s13068-018-1209-6 (PMC6062969; doi:10.1186/s13068-018-1209-6)
Supplement: Supplementary file 5 — Additional file 5: Figure S4. Hydrolysis of un/pretreated wheat straw. The wild-type PC9 and the two transformants were used as a biological pretreatment of wheat straw for 19 and 35 days. The pretreated and untreated straw samples were hydrolyzed by the commercial cellulase cocktail Cellic® CTec2 (Novozymes), and the concentration of released soluble sugars was measured by the DNS method [84]. [file 13068_2018_1209_MOESM5_ESM.docx]

**Additional file 5**

PC9

OE*cre1*

KO*cre1*

None

**Figure S4. Hydrolysis of un/pretreated wheat straw.** The wild type PC9 and the two transformants were used as a biological pretreatment of wheat straw for 19 and 35 days. The pretreated and untreated straw samples were hydrolyzed by the commercial cellulase cocktail Cellic® CTec2 (Novozymes), and the concentration of released soluble sugars was measured by the DNS method [1].

1. Miller GL. Use of dinitrosalicylic acid reagent for determination of reducing sugar. Anal Biochem. 1959;31:426–8.
