# Supplementary material for: Effects of cre1 modification in the white-rot fungus Pleurotus ostreatus PC9: altering substrate preference during biological pretreatment
Source: Biotechnol Biofuels. 2018 Jul 27;11:212. doi: 10.1186/s13068-018-1209-6 (PMC6062969; doi:10.1186/s13068-018-1209-6)
Supplement: Supplementary file 6 — Additional file 6: Table S2. Oligonucleotides used in this study. The oligonucleotides that are used for either construction of cassettes or verification of the genomic integration of both OEcre1 or KOcre1 are detailed in the table. The location of the primer within the cassettes or in the genome is given in Additional file 1: Figure S1a and b. [file 13068_2018_1209_MOESM6_ESM.docx]

**Additional file 6**

**Table S2**. **Oligonucleotides used in this study.** The oligonucleotides that were used for either construction of cassettes or verification of the genomic integration of both OE*cre1* or KO*cre1* are detailed in the table. The location of the primer within the cassettes or in the genome is given in Fig. 1

| Template | Target | Primer designation | Sequence (5'→3') | Expected amplicon (bp) |
| --- | --- | --- | --- | --- |
| **Cassette construction** | | | | |
| *cre1* over-expression cassette (TMS17) | | | | |
| PC9 genomic DNA | *beta-tubulin* | SacII-btubPF | CCGCGGATGCTGTTGGGAGGAAACTAAAT | 1704 |
|  | *beta-tubulin* | cre1F-btubPR | CGACTAACTTCTTTTCCATGCAGAATGCCCTCCTCGTCCCAG |  |
| PC9 genomic DNA | *cre1* | btubPR-cre1F | CTGGGACGAGGAGGGCATTCTGCATGGAAAAGAAGTTAGTCG | 3614 |
|  | *cre1* | cre1R-SphI | TCACGTACGATGAAACGACTGCATGC |  |
| *cre1* replacement cassette (TMS18) | | | | |
| PC9 genomic DNA | *cre1* | cre1PF | GCCCCTGCCCCTATGCT | 2023 |
|  | *cre1* | hygF-cre1PR | ATTTAGTTTCCTCCCAACAGCATGGCGGTGGTGGTGGTG |  |
| pTMS14 (see reference xx) | *Hyg^r^* | cre1PR-hygF | CACCACCACCACCGCCATGCTGTTGGGAGGAAACTAAAT | 3204 |
|  | *Hyg^r^* | cre1TF-hygR | CGTAAGAAGAGAAAGAGGCGGAATGTTGGTCAGCGCTGTGG |  |
| PC9 genomic DNA | *cre1* | hygR-cre1TF | CCACAGCGCTGACCAACATTCCGCCTCTTTCTCTTCTTACG | 2019 |
|  | *cre1* | cre1TR | CGAACACAACCTGCAATATGCCT |  |
| **Analysis of construct integration** | | | | |
| *cre1* over-expression cassette (TMS17) | | | | |
| strain-specific genomic DNA | *Cbx^r^* | TMS17DF | CGGGAACACACAAATCATTG | 6147 |
|  | *cre1* | TMS17DR | GCTGTCCAAAAGAGGGTGTC |  |
| *cre1* replacement cassette (TMS18) | | | | |
| strain-specific genomic DNA | *cre1* | cre1DF | TCCTTTAAGTGTGTGCCGCT | 3589 |
|  | *cre1* | cre1DR | CTGCGGCACTGAAACCAAAA |  |
