# Supplementary material for: Effects of cre1 modification in the white-rot fungus Pleurotus ostreatus PC9: altering substrate preference during biological pretreatment
Source: Biotechnol Biofuels. 2018 Jul 27;11:212. doi: 10.1186/s13068-018-1209-6 (PMC6062969; doi:10.1186/s13068-018-1209-6)
Supplement: Supplementary file 7 — Additional file 7: Figure S5. cre1 gene expression analysis. PC9 and OEcre1 were grown for 7 days on minimal media containing either CMC or glucose as a carbon source. Culture biomass was ground under liquid nitrogen with mortar and pestle, and RNA was purified by RNeasy Plus Mini Kit (Qiagen) according to the manufacturer’s instructions. cDNA was synthesized using the qScript cDNA Synthesis Kit (Quanta BioSciences, Gaithersburg, MD, USA). Quantification of transcript abundance was determined on an ABI StepOnePlus Real-Time PCR Sequence Detection System and software (Applied Biosystems), using Power SYBR Green PCR Master Mix (Applied Biosystems), with an annealing temperature of 63 °C, according to the manufacturer’s default operating procedures. The endogenous internal control gene used was β-tubulin. Primers tubF543 (5′-GTGCGTAAGGAAGCTGAGGG-3′) and tubR777 (5′-TGTGGCATTGTACGGCTCAAC-3′) were used to amplify a 201-bp amplicon from β-tubulin. Primers cre1F_2214 (5′-GTGGATTGGGCGGGTCGA-3′) and cre1R_2623 (5′-CATCCGTTCCCATGAGCGAT-3′) were used to amplify a 201-bp amplicon from cre1. Target gene transcript abundance is expressed relative to the levels of β-tubulin according to their Ct values 2 [(Ct β−tubulin) − (Ct cre1)], in arbitrary units. [file 13068_2018_1209_MOESM7_ESM.docx]

Additional file 7.

Expression levels relative to β-tubulin

**Figure S5. *cre1* gene expression analysis.** PC9 and OE*cre1* strains were grown for seven days on minimal media containing either CMC or glucose as a carbon source. Culture biomass was ground under liquid nitrogen with mortar and pestle, and RNA was purified by RNeasy Plus Mini Kit (Qiagen) according to the manufacturer’s instructions. cDNA was synthesized using the qScript cDNA Synthesis Kit (Quanta BioSciences, Gaithersburg, MD, USA). Quantification of transcript abundance was determined on an ABI StepOnePlus Real-Time PCR Sequence Detection System and software (Applied Biosystems), using Power SYBR Green PCR Master Mix (Applied Biosystems), with an annealing temperature of 63°C, according to the manufacturer’s default operating procedures. The endogenous internal control gene used was *β-tubulin*. Primers tubF543 (5’-GTGCGTAAGGAAGCTGAGGG-3’) and tubR777 (5’-TGTGGCATTGTACGGCTCAAC-3’) were used to amplify a 201-bp amplicon from *β-tubulin*. Primers cre1F_2214 (5’-GTGGATTGGGCGGGTCGA-3’) and cre1R_2623 (5’-CATCCGTTCCCATGAGCGAT-3’) were used to amplify a 201-bp amplicon from *cre1*. Target gene transcript abundance is expressed relative to the levels of *β-tubulin* according to their Ct values 2 ^[(Ct β-tubulin) - (Ct^*^cre1^*^)]^, in arbitrary units.
